# Conium

**Published:** 1897-02

**Authors:** 


THE
### HOMŒOPATHIC PHYSICIAN,
A MONTHLY JOURNAL OF
HOMŒOPATHIC MATERIA MEDICA AND CLINICAL MEDICINE.

"If our school ever gives up the strict inductive method of Hahnemann, we are lost, and deserve only to be mentioned as a caricature in the history of medicine."—CONSTANTINE HERING.

Vol. XVII. FEBRUARY, 1897. No. 2.

### EDITORIAL.

CONIUM.—There is very little to be added from the comments of Dr. Lippe to what has been already said of Conium.

These comments are as follow:

Induration of the parotid gland after contusion.

Cancer of the lips following a contusion. Cancer of the lips from the pressure of the stem of a pipe in smoking. This may be regarded as a kind of contusion.

Involuntary deglutition when walking in the wind. This is Dr. Guernsey's keynote.

This reminds us of a few wind symptoms which any one may meet with in the materia medica, but which none have collected together for instant reference.

Cold wind causes dull colicky pain around the navel, Kali-bichrom.

Walking in rough wind causes pain in left occipital protuberance, Muriatic-acid.

Walking against the wind aggravates hoarseness, Nux-moschata.

Walking against the wind causes sensation of soreness in the brain, China or Cinchona.

Riding in cold wind causes aggravation, Rumex-crispus.

Walking against the wind causes stoppage of breathing, Calcarea-carb.

Great dread of wind, Chamomilla.

Wind causes aggravation of ulcers, Chamomilla.

Walking in cold wind causes headache, Muriatic-acid.

Riding against a cold west wind caused cold in the head, followed by croup, Kali-bichromicum.

Windy weather causes increase of dyspnœa, even when well protected and in a warm room, Arsenicum.

Under Conium there is alternate flowing and stopping of urine. This is one of its great characteristics. It is Dr. Guernsey's keynote.

Dr. Lippe related a case of diabetes in which there was nine per cent. of sugar present in the urine. The patient discharged immense quantities of urine. There was at the same time swelling and suppuration of the left parotid gland. The patient would waken from sleep in a profuse perspiration and would be unable to fall to sleep again. The perspiration stood up in great beads and saturated the bed-clothes. In this case the foregoing symptoms were perfectly relieved, though Dr. Lippe did not expect the patient to get well. The remedy was given in the thirtieth potency in water, and as he was in extreme danger it was continuously administered for a day or two. Later, when improvement began to flag he was given a dose of the two hundredth potency. He was thus kept up for over two weeks, and when he then began to sink a dose of the seventy thousandth potency was given. This sustained him for awhile, but ultimately he died. In critical cases, when the properly-selected remedy fails to benefit the patient for more than two or three days, we may reckon upon certain death. If, however, its beneficial action last more than a week or continue several weeks, we may look for his ultimate cure.

Conium has bleeding of the gums like Mercurius and Carbo-vegetabilis.

Conium has heartburn, beginning as soon as the patient lies down. The burning comes up the throat and compels him to get up.

This is another of Dr. Guernsey's keynotes.

Conium has spasmodic pains in the stomach, and this is similar to Calcarea-carbonica and Silicea.

Conium has swelling of the testes from contusion. Pulsatilla also has swelling of the testicles.

Conium has dysmenorrhœa, with sharp pains about the heart.

Conium has loose cough with inability to expectorate. He must swallow what is loosened by coughing. Causticum and Kali-carbonicum have this loosening of phlegm which cannot be raised.

Conium has red spots on the calves, turning yellow or green as if from contusion. Arnica has this symptom also.

Conium catches cold from slight exposure of the feet. This is similar to Silicea.

Dr. Guernsey's keynotes are the following:

Violent itching of the vulva followed by pressing down of the uterus; the urine flowing and stopping at every emission. Violent itching after the menses. Induration from injuries. Large pimples on the mons veneris, painful to the touch. Cutting pain between the labia on micturition. Severe stitches in the vulva. Violent itching of the vulva. Shriveling of the mammary glands and increased sexual desire. Much trouble with vertigo when lying down and turning over in bed. Urine flows and stops and flows again at each emission. Burning, sore, aching sensation in the uterus. Bitter taste in the mouth and thirst. Pulse unusually slow, or intermitting. Leucorrhœa of white acrid mucus causing burning, smarting sensation. Leucorrhœa like milk, painful and excoriating with induration and prolapsus uteri at the same time. Breasts become sore, enlarged, and painful at every menstrual period. Prolapsus complicated with induration, ulceration, and profuse leucorrhœa. Suitable to women of tight rigid fibre and easily excited. Stitches in the vagina and pressing from above downward. Dysmenorrhœa with shooting pains in left side of the chest. Drawing in the legs during the menses. Painful abdominal spasms. At every menstrual period the breasts become large, sore, and painful. Pricking, stinging pains. Is aroused from sleep by the pains. Induration and enlargement of the ovary with nausea, vomiting, eructations of wind and expectoration of phlegm. Lancinating pains. Acrid, white, slimy leucorrhœa. Labor-like contractions. Pains in the iliac regions. Stitches from abdomen to the right side of chest. Constant and ineffectual urging to stool. Globus hystericus. Heat and burning in the rectum during stool, and tremulous weakness afterward. Stitches in the anus between the stools. Frequent and ineffectual urging to stool, or a small quantity only each time. Stinging in neck of uterus. Scirrhus of any part. Terrible nausea and vomiting in women who have scirrhus nodes during pregnancy. Spasmodic labor pains with rigidity of os uteri. Hardness and distention of abdomen with frequent sour eructations. Violent fits of coughing, mostly during the night. Parotid and submaxillary glands are swollen and hard as a stone. Lips and teeth covered with black crusts. The patient has hot skin and is delirious.

